# Hepatic zonation of carbon and nitrogen fluxes derived from glutamine and ammonia transformations

**DOI:** 10.1186/1423-0127-17-1

**Published:** 2010-01-07

**Authors:** Jurandir F Comar, Fumie Suzuki-Kemmelmeier, Jorgete Constantin, Adelar Bracht

**Affiliations:** 1 Laboratory of Liver Metabolism, Biochemistry Department, University of Maringá, Maringá, Brazil

## Abstract

**Background:**

Glutaminase predominates in periportal hepatocytes and it has been proposed that it determines the glutamine-derived nitrogen flow through the urea cycle. Glutamine-derived urea production should, thus, be considerably faster in periportal hepatocytes. This postulate, based on indirect observations, has not yet been unequivocally demonstrated, making a direct investigation of ureogenesis from glutamine highly desirable.

**Methods:**

Zonation of glutamine metabolism was investigated in the bivascularly perfused rat liver with [U-^14^C]glutamine infusion (0.6 mM) into the portal vein (antegrade perfusion) or into the hepatic vein (retrograde perfusion).

**Results:**

Ammonia infusion into the hepatic artery in retrograde and antegrade perfusion allowed to promote glutamine metabolism in the periportal region and in the whole liver parenchyma, respectively. The results revealed that the space-normalized glutamine uptake, indicated by ^14^CO_2 _production, gluconeogenesis, lactate production and the associated oxygen uptake, predominates in the periportal region. Periportal predominance was especially pronounced for gluconeogenesis. Ureogenesis, however, tended to be uniformly distributed over the whole liver parenchyma at low ammonia concentrations (up to 1.0 mM); periportal predominance was found only at ammonia concentrations above 1 mM. The proportions between the carbon and nitrogen fluxes in periportal cells are not the same along the liver acinus.

**Conclusions:**

In conclusion, the results of the present work indicate that the glutaminase activity in periportal hepatocytes is not the rate-controlling step of the glutamine-derived nitrogen flow through the urea cycle. The findings corroborate recent work indicating that ureogenesis is also an important ammonia-detoxifying mechanism in cells situated downstream to the periportal region.

## Background

Glutamine is one of the most abundant amino acids in the organism of mammals and it is involved in more metabolic processes than any other amino acid [1]. Also for the liver the role of glutamine is very important. It is known that the metabolism of glutamine presents zonation [2], i.e., the different regions along the hepatic acini respond in a different way to the amino acid [3-5]. The underlying mechanisms of the zonation of enzymes involved in glutamine metabolism are controversial. Recent work has suggested a role for levels of substrate, autocrine soluble factor or cytoskeleton interactions putatively associated with the beta-catenin signaling pathway [6]. Knowledge about zonation of the metabolism of L-glutamine is centered mainly on nitrogen metabolism. The dominant idea is that, along the hepatic acinus, the pathways of urea production and glutamine synthesis are arranged in sequence in order to optimize ammonia detoxification. The urea synthesis in the periportal region represents the system of low affinity for ammonia detoxification. Glutamine synthesis in the perivenous zone represents the system of high affinity for ammonia detoxification. The periportal glutaminase [7], located in the mitochondria, is stimulated by ammonia and influenced by pH and hormones [8,9]. The activity of this enzyme is believed to determine, partly at least, the flow of nitrogen derived from glutamine through the urea cycle [10]. The glutamine synthetase, restricted to a limited number of perivenous hepatocytes, is believed to act as a kind of scavenger for the ammonia that escapes from the periportal urea synthesis [3].

If the activity of glutaminase determines the nitrogen flow derived from glutamine through the urea cycle, urea production from glutamine should be considerably faster in periportal hepatocytes [5,10]. This postulate is based on the measurement of enzymatic activities under artificial conditions and has not yet been unequivocally demonstrated by flux measurements in intact cell systems. Such measurements are important, however, because discrepancies between enzyme activity or gene expression evaluations and the actual metabolic fluxes in the living cell are common. Recent studies have shown, for example, that urea production from alanine, lactate + ammonia and pyruvate + ammonia is faster in cells situated downstream to the periportal zone at most substrate concentrations [11,12] in spite of the observation that the expression of key enzymes from the urea cycle predominates in these cells [13]. Periportal predominance of urea production was found only at high ammonia concentrations in the presence of pyruvate [12]. Absence of correlation between enzyme activity or enzyme expression and metabolic fluxes in the cell are actually quite common and direct measurements of the latter are, thus, desirable. Moreover, glutamine is also a gluconeogenic substrate and it has been found that periportal and perivenous cells present different glucose to urea production ratios from alanine [11]. This is an important observation if one takes into account the reciprocal regulation of both ureogenesis and gluconeogenesis [14,15], which seems to be different in periportal and perivenous cells, and raises the question about the relative proportions between ureogenesis and glutamine transformation. These and other questions prompted us to undertake a detailed investigation of the zonation of glutamine transformation with the simultaneous measurement of nitrogen and carbon fluxes. The methodology to be utilized is the bivascularly perfused rat liver, which allows to reach selectively periportal hepatocytes via the hepatic artery in retrograde perfusion [16] and which has been successfully used for investigating hepatic zonation without significant alterations of the liver structure [11,12,17].

## Methods

### Materials

The liver perfusion apparatus was built in the workshops of the University of Maringá. Enzymes and coenzymes used in the metabolite assays were purchased from Sigma Chemical Co. (St Louis, USA). [U-^14^C]Glutamine (258 mCi/mol) was purchased from Amersham Bioscience (Buckimghamshire, UK). All standard chemicals were from the best available grade (>99.5% purity) and were purchased from Merck (Darmstadt, FRG), Carlo Erba (São Paulo, Brasil) and Reagen (Rio de Janeiro, Brazil).

### Animals and bivascular liver perfusion

Male albino rats (Wistar), weighing 180-220 g, were fed *ad libitum *with a standard laboratory diet (Purina^®^). Food was withdrawn 18 hours prior to the liver perfusion experiments. For the surgical procedure of liver isolation, the rats were anesthetized by intraperitoneal injection of sodium pentobarbital (50 mg/kg). All experiments were done in accordance with the world-wide accepted ethical guidelines for animal experimentation. The authors were duly authorized by the Coordination of the PhD Program in Biological Sciences of the University of Maringá to conduct this animal study.

**Figure 1:**
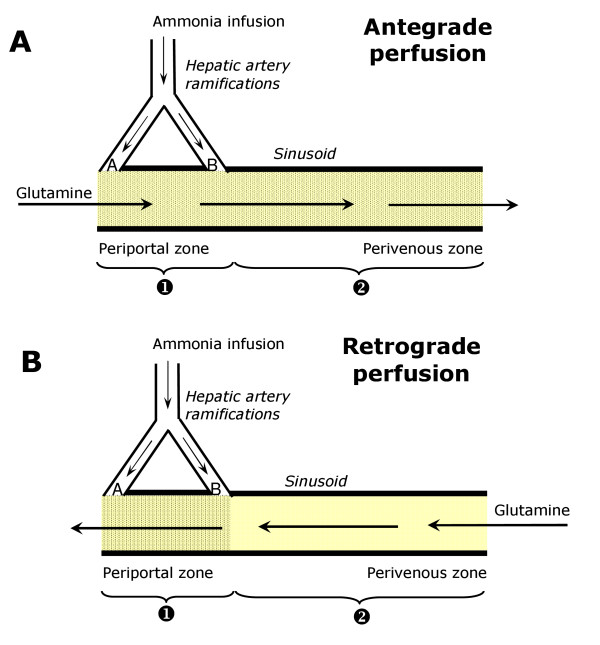
**Schematic representation of some characteristics of the hepatic microcirculation and the experimental protocols**. The arrows indicate the direction of flow. Legends: **A **the presinusoidal confluence of the arterial and portal bed; **B **the intrasinusoidal confluence of the arterial and portal bed.

Hemoglobin-free, non-recirculating bivascular liver perfusion was performed either in the antegrade mode (entry via the portal vein plus hepatic artery and exit via the hepatic vein) or in the retrograde mode (entry via the hepatic vein plus hepatic artery and exit via the portal vein; Fig. 1). The surgical procedure was described previously [17]. *In situ *perfusion was carried out, the flow being provided by two peristaltic pumps. The perfusion fluid was Krebs/Henseleit-bicarbonate buffer (pH 7.4) containing 25 mg% bovine-serum albumin, saturated with a mixture of oxygen and carbon dioxide (95:5) by means of a membrane oxygenator with simultaneous temperature adjustment (37°C). The portal flow was adjusted between 28 and 32 ml/min and the arterial flow between 2 and 3 ml/min. All perfusion experiments were initiated in the antegrade mode. Retrograde perfusion was established by changing the direction of flow at 15-20 minutes before initiating sampling of the effluent perfusate. In all perfusion experiments, livers from fasted rats were used so that glycogenolysis and glycolysis from endogenous sources was minimal [18].

### Analytical

Samples of the effluent perfusion fluid were collected according to the experimental protocol and analyzed for their metabolite contents. The following compounds were measured by means of standard enzymatic procedures: glucose, lactate, urea and ammonia [19]. The oxygen concentration in the outflowing perfusate was monitored continuously, employing a teflon-shielded platinum electrode adequately positioned in a plexiglass chamber at the exit of the perfusate [20].

The carbon dioxide production from L-[U-^14^C]glutamine was measured by trapping ^14^CO_2 _in phenylethylamine [21]. Radioactivity was measured by liquid scintillation spectroscopy. The following scintillation solution was used: toluene/ethanol (2/1) containing 5 g/liter 2,5-diphenyloxazole and 0.15 g/liter 2,2-p-phenylene-bis(5-phenyloxazole).

### Experimental protocol and data analysis

The experimental protocol that was adopted in the present work takes advantage of some particularities of the hepatic microcirculation of the rat. In bivascular perfusion of the rat liver (Fig. 1), a portion of the periportal hepatic parenchyma can be reached selectively with substances that are infused into the hepatic artery in retrograde perfusion [11,12,16]. In the present work glutamine was infused at the physiological concentration of 0.6 mM into the portal vein in antegrade perfusion and into the hepatic vein in retrograde perfusion so that all hepatocytes were supplied with this substrate. At the concentration of 0.6 mM, however, metabolic transformation of glutamine only occurs when ammonia is present in the perfusion fluid [8]. In the present work the latter was infused into the hepatic artery. By virtue of the existence of two arterio-sinusoidal confluences, if ammonia is infused into the hepatic artery in retrograde perfusion only periportal hepatocytes will be supplied with this compound (Fig. 1B) [11,12,16]. Consequently, only periportal cells will be stimulated to metabolize glutamine. If ammonia is infused into the hepatic artery in antegrade perfusion, however, all hepatocytes will be supplied (Fig. 1A) and also all hepatocytes will transform glutamine [11,12,16]. Comparison of the metabolic fluxes in antegrade (J_ant_) and retrograde (J_ret_) perfusion requires normalization by dividing them through the corresponding accessible cell spaces, as determined by previous work [22,23]. Analysis also requires to take into account the different concentrations of ammonia in the regions that are accessible during antegrade and retrograde perfusion, which depend on the distribution of the arterial flow between the pre- and intrasinusoidal confluences (Fig. 1). A simple mathematical description of the flux responses that can be expected when substrates and effectors are infused into the hepatic artery in antegrade or retrograde perfusion has been proposed in previous work [11,12,24]. The same treatment can be extended to the situation in which a metabolic effector such as ammonia is infused. If J_ret _and J_ant _are the cell space-normalized metabolic responses of glutamine metabolism due to the ammonia infusion into the hepatic artery in retrograde and antegrade perfusion (Fig. 1), respectively, the following relations are valid: [11,12,24]:(1)

In equation (1) v_1R _is the metabolic flux in region 1 in retrograde perfusion (Fig. 1B), function (*f*) of the ammonia concentration (c_1R_) in this region during retrograde perfusion, whereas v_1A _is the metabolic flux in region 1 in antegrade perfusion, function of the ammonia concentration (c_1A_) in this region in antegrade perfusion. The values of c_1R _and c_1A _are not the same even though the difference is not very pronounced [12]. They are different for a given ammonia infusion rate because the arterial flow that reaches the intrasinusoidal confluence (F_AIS_) corresponds to 58% of the total arterial flow (F_A_), i.e., F_AIS _= 0.58F_A _[12]. The symbol v_2A _represents the metabolic flux in region 2, also function of the ammonia concentration (c_2A_) during antegrade perfusion (Fig. 1A). The parameter r is the fraction of the cellular space that can be reached via the hepatic artery in retrograde perfusion; this parameter has been measured previously as being equal to 0.38 in the hemoblogin-free perfused rat liver [22,23].

Comparison of the v_1R _versus c_1R _and v_2A _versus c_2A _relationships should provide an immediate answer about a possible different sensitivity of glutamine metabolism to ammonia in regions 1 and 2, v_1R _= v_2A _meaning absence of zonation, v_1R _> v_2A _periportal predominance and v_1R _< v_2A _perivenous predominance. It should be noted, however, that when J_ret _> J_ant _is observed for a given infusion rate, this can be immediately interpreted as periportal predominance in all cases where a positive correlation exists between fluxes and concentrations, because c_2A _is always greater than c_1R _for identical infusion rates [12].

The value of v_1R _corresponds to J_ret _as given by equation (1). Values of c_1R _were calculated using the relation [24](3)

where  is the rate of ammonia infusion (μmol min^-1 ^g liver^-1^), F_T _the total flow through the liver (ml min^-1^), F_A _the arterial flow (ml min^-1^) and F_AIS _the arterial flow that reaches the intrasinusoidal confluence (F_AIS _= 0.58F_A_) [12]. Following c_1R _calculation, v_1A _can be obtained by interpolating within the v_1R _versus c_1R _curve. Using these v_1A _values, v_2A _can be calculated using the relation(4)

derived from Eq. (2). The c_2A _values, finally can be determined simply as [24](5)

### Treatment of data

Statistical analysis of the data was done by means of the Statistica™ program (Statsoft^®^, 1998). The Scientist software from Micro Math Scientific Software (Salt Lake City, UT) was used for the numerical interpolations (using Stineman's interpolation formula).

## Results

### Time courses of the reponses to glutamine and ammonia infusions

**Figure 2:**
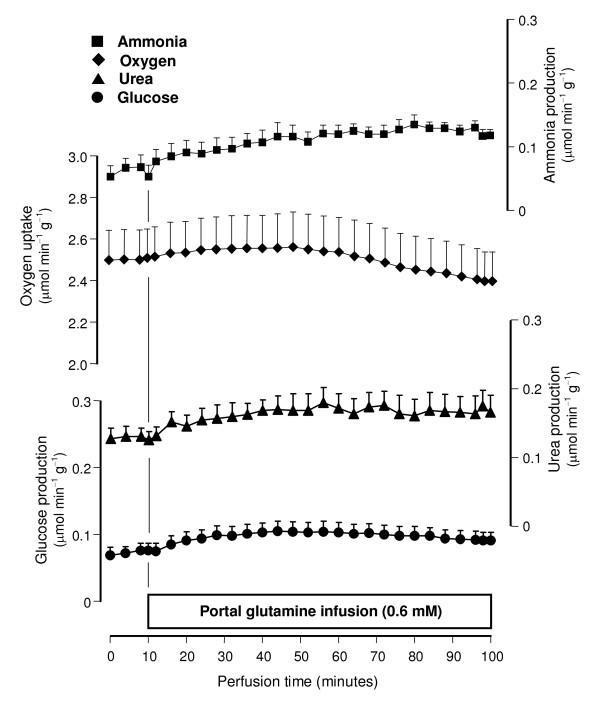
**Time courses of ammonia, urea and glucose productions and oxygen uptake during portal infusion of 0.6 mM glutamine**. Livers from fasted rats were perfused as described in Materials and Methods. Glutamine was infused as indicated. Data are from 4 liver perfusion experiments and error bars are mean standard errors.

**Figure 3:**
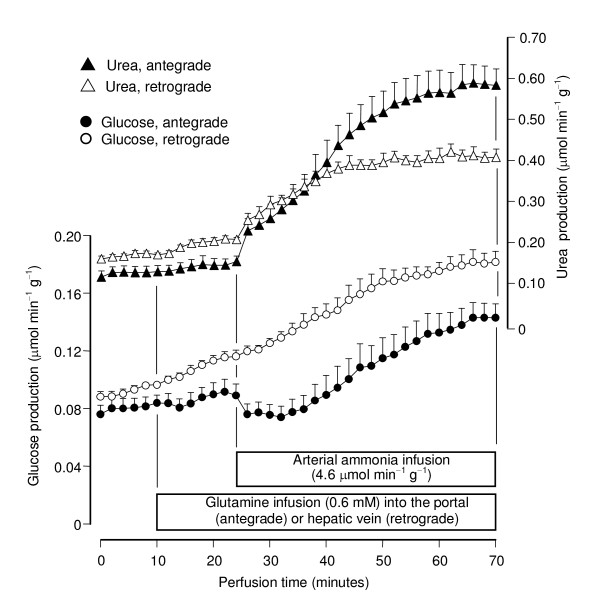
**Time courses of the actions of arterially infused ammonia on urea and glucose productions from [U-^14^C]glutamine in antegrade and retrograde perfusion**. Livers from fasted rats were perfused as described in Materials and Methods. [U-^14^C]Glutamine and ammonia were infused as indicated. Data are from 5 liver perfusion experiments and error bars are mean standard errors.

**Figure 4:**
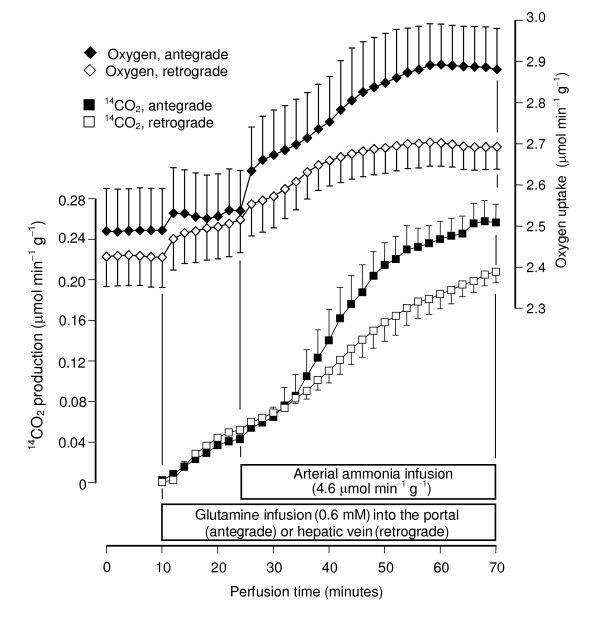
**Time courses of the actions of arterially infused ammonia on ^14^CO_2 _production from [U-^14^C]glutamine and the corresponding oxygen uptake increments in antegrade and retrograde perfusion**. Livers from fasted rats were perfused as described in Materials and Methods. [U-^14^C]Glutamine and ammonia were infused as indicated. Data are from 5 liver perfusion experiments and error bars are mean standard errors.

All perfusion experiments were done with livers from 18 hours fasted rats in order to minimize interference by glycogen catabolism. Control experiments (Fig. 2) confirmed the earlier observations that the infusion of glutamine at physiological concentrations (0.6 mM) does not produce significant increases in glucose, urea and ammonia productions and oxygen uptake even if the infusion time is as long as 90 minutes [8]. Ammonia infusion into the hepatic artery, however, increased glutamine metabolism in both antegrade and retrograde perfusion (Figs. 3 and 4). Five parameters were measured: urea, lactate, glucose and ^14^CO_2 _productions and oxygen consumption. Pyruvate production, which is significant at high glutamine concentrations (2.5 mM; [25]), was not significantly increased at the low physiological glutamine concentrations employed in the present work. Both glucose and urea productions increased significantly after initiation of ammonia infusion either in antegrade or retrograde perfusion (Fig. 3). At 70 minutes perfusion time new steady-state levels were reached. Notably, glucose production in antegrade perfusion experienced a transient decline just after initiation of ammonia infusion before elevating to a new steady-state. Also notable is the observation that glucose production in retrograde perfusion was higher than that in antegrade perfusion in spite of the fact that a considerably smaller cell space is reached by arterially infused ammonia in retrograde perfusion when compared to antegrade perfusion (Fig. 1). Oxygen uptake and ^14^CO_2 _production during portal or venous [U-^14^C]glutamine infusion were also increased by arterially infused ammonia in both antegrade and retrograde perfusion (Fig. 4). They had a similar tendency of reaching state-state levels at the end of the experiment. Note that there are relatively small, though significant, ^14^CO_2 _productions even in the absence of ammonia, a phenomenon that was also observed in earlier work [8]. They are equal in antegrade and retrograde perfusion, however, reflecting the fact that glutamine infused into either the portal or hepatic veins has access to the same cell masses. As expected, basal oxygen uptake rates in antegrade and retrograde perfusion were also nearly the same.

### Cell space corrected fluxes of glutamine metabolism

So far the metabolic fluxes were expressed as μmol min^-1 ^g^-1 ^(Figs. 3 and 4). This is valid for comparing basal rates or rates before ammonia infusion because glutamine, when infused into the portal or hepatic veins, has access to the whole liver parenchyma. In order to compare the ammonia-dependent glutamine metabolism, however, the metabolic fluxes must be normalized with reference to the cell spaces that are accessible via the hepatic artery in antegrade and retrograde perfusion. These spaces are equal to 0.684 and 0.266 ml/g, respectively, for antegrade and retrograde perfusion [22,23]. For the normalization process the rates before ammonia infusion were subtracted from the rates at the end of the ammonia infusion period (70 minutes perfusion time in Figs. 3 and 4); this difference was than divided by the corresponding accessible cell space. Implicit in this procedure is the assumption that the increments caused by ammonia reflect solely the contribution of the cell spaces that are accessible via the hepatic artery in antegrade and retrograde perfusion, whereas the basal rates represent the contribution of the whole liver parenchyma (Fig. 1). The normalized fluxes calculated in this way correspond to the variables J_ret _and J_ant _as defined by equations (1) and (2), respectively.

It is apparent (Table 1) that the ammonia-dependent fluxes were faster in retrograde perfusion (i.e., J_ret _> J_ant_) for all ammonia infusion rates with the exception of urea production. The most accentuated difference was found for glucose production, but the difference was also significant for ^14^CO_2 _production and oxygen uptake. As mentioned and justified in the Materials and Methods section the observation J_ret _> J_ant _always means an enrichment of the metabolic activity in the periportal space accessible via the intrasinusoidal confluence in comparison to the mean metabolic activity of the liver.

**Table 1:** Changes in glucose,^14^CO_2 _and urea productions and oxygen uptake due to glutamine + ammonia as a function of the arterial ammonia infusion in antegrade and retrograde perfusion.

Metabolic parameter	Ammonia infusion (μmol min^(1 ^g^(1^)	J_ant_(μmol min^(1 ^ml^(1^)	J_ret_(μmol min^(1 ^ml^(1^)	***p***
Extra glucose production	1.25 4.6 9.2	0.052 ± 0.004 (n = 4) 0.101 ± 0.011 (n = 4) 0.165 ± 0.018 (n = 5)	0.137 ± 0.022 (n = 4) 0.251 ± 0.036 (n = 4) 0.303 ± 0.040 (n = 4)	0.002 0.007 0.011

Extra lactate production	1.25 4.6 9.2	0.038 ± 0.006 (n = 4) 0.045 ± 0.010 (n = 4) 0.055 ± 0.008 (n = 5)	0.048 ± 0.014 (n = 5) 0.112 ± 0.023 (n = 5) 0.086 ± 0.008 (n = 5)	0.57 0.045 0.025

Extra oxygen uptake	1.25 4.6 9.2	0.284 ± 0.045 (n = 4) 0.524 ± 0.040 (n = 4) 0.815 ± 0.076 (n = 5)	0.451 ± 0.069 (n = 5) 0.877 ± 0.136 (n = 4) 1.090 ± 0.070 (n = 5)	0.098 0.047 0.029

^14^CO_2 _production	1.25 4.6 9.2	0.182 ± 0.037 (n = 3) 0.318 ± 0.028 (n = 4) 0.545 ± 0.046 (n = 5)	0.425 ± 0.067 (n = 5) 0.611 ± 0.040 (n = 5) 0.886 ± 0.058 (n = 5)	0.041 0.001 0.002

Extra urea production	1.25 4.6 9.2	0.496 ± 0.046 (n = 4) 0.702 ± 0.028 (n = 3) 0.970 ± 0.078 (n = 5)	0.421 ± 0.067 (n = 5) 0.663 ± 0.056 (n = 4) 1.117 ± 0.059 (n = 5)	0.41 0.67 0.17

The raw data were obtained from experiments of the type illustrated by Figures 3 and 4. The glutamine concentration was the same for all experiments, namely 0.6 mM. The changes correspond to the final metabolic fluxes in the presence of both glutamine and ammonia (70 minutes perfusion time in Figures 3 and 4), subtracted from the fluxes in the sole presence of glutamine. All values were expressed as μmol per minute per ml cell space that is accessible via the hepatic artery in each perfusion mode. The data were analyzed by variance analysis. The *p *values were obtained by applying the Student-Newman-Keuls test.

### Fine analysis of the zonation of glutamine metabolism

**Figure 5:**
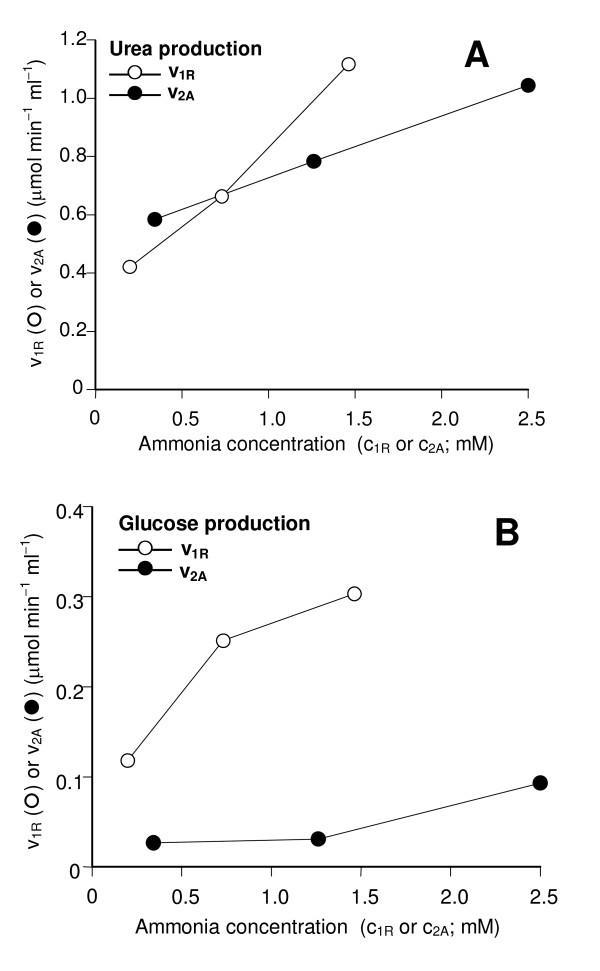
**Urea and glucose productions from glutamine and ammonia in 2 different zones along the hepatic parenchyma as a function of the extracellular ammonia concentrations**. The corresponding experimental data J_ant _and J_ret _in Table 1 were used to calculate v_1R _and v_2A _using equations (3) to (5) and the numerical procedures described in the text; c_1R _and c_2A _were calculated from the rates of ammonia infusion, the total flow and the arterial flow as described in the text.

The near equality of J_ant _and J_ret _for urea production from glutamine (Table 1), needs a more accurate analysis if one wishes to conclude about the distribution along the hepatic acinus. The analysis can be done using equations (1), (3), (4) and (5) for calculating the metabolic fluxes in the different spaces that are accessible via the hepatic artery in antegrade and retrograde perfusion, namely v_1R_, v_2A _, c_1R _and c_2A_. The v_1R _versus c_1R _and v_2A _versus c_2A _plots (Fig. 5A) reveal that urea production in region 1 (v_1R_; periportal cells) was not superior to that in region 2 (v_2A_; mainly perivenous cells) at low ammonia concentrations. Only for concentrations above 1 mM periportal predominance of ureogenesis can be expected. In region 2 urea production was apparently near to saturation in the range between 1 and 2 mM. In the periportal region, however, no saturation was evident in the range up to 1.5 mM. When the same analysis that was done for urea production was repeated for glucose production, the results fully confirmed the conclusion that gluconeogenesis from glutamine amply predominates in periportal cells (Fig. 5B), as revealed by the great difference in gluconeogenesis from glutamine in regions 1 and 2. Actually, for low ammonia concentrations there was almost no glucose production in the liver cells situated downstream to the intrasinusoidal confluence of the arterial and portal beds (Fig. 5B).

## Discussion

The main question of the present study was related to the accepted notion that the ammonia-dependent glutaminase in periportal hepatocytes determines the glutamine-derived nitrogen flow through the urea cycle. This would imply in a clear predominance of ureogenesis in periportal cells because this enzyme is much more concentrated in these cells [7]. Our results did not confirm this hypothesis for low and physiologic ammonia concentrations. Actually, for low ammonia concentrations there was a clear tendency toward lower rates of urea production in periportal cells. Periportal predominance was found only at high ammonia concentrations. In this respect it is noteworthy to mention that in previous experiments, conducted with lactate + ammonia and pyruvate + ammonia as precursors of glucose and urea, predominance of urea production in cells localized downstream to the periportal region was found for most conditions [12]. The exception was the condition pyruvate + high ammonia concentrations, for which periportal predominance of ureogenesis was found. All these observations are somewhat surprising because periportal predominance of the activity and expression of key enzymes of the urea cycle has been found in several studies [13,26]. It is also true, however, that the enzymes of the urea cycle are present in 93 to 95% of the parenchymal cells. The key-enzyme carbamoyl-phosphate synthase, for example, seems to be absent only from those hepatocytes immediately surrounding the hepatic venules [26]. The hepatocytes not containing carbamoyl-phosphate synthase are precisely those ones containing glutamine synthetase, which have been estimated as comprising only 5 to 7% [27]. Most hepatocytes, thus, are perfectly able to synthesize urea and the final ureogenic activity will depend not only on the maximal activity (which is that one detected when "enzyme activities" are measured) but also on the real cellular conditions in terms of the concentrations of substrates and allosteric regulators. A question that can be raised at this point is about the factors that limit ureogenesis in the periportal region in spite of the potentially higher enzyme activities [26,27]. With the available data only hypothetical possibilities can be discussed. The production of N-acetyl-glutamate, the key activator of carbamoyl-phosphate synthase [28] should not be restrained by the periportal availability of glutamate, which is the immediate product of glutamine deamination. It should be remembered, however, that the N-acetyl-glutamate synthase is itself a regulatory enzyme, dependent on regulatory mechanisms with participation of arginine [29] and ornithine [30] whose concentrations are not necessarily equal along the hepatic acinus. Aspartate is an essential amine group donnor for the urea cycle and it is produced by the aspartate transaminase reaction. This enzyme is said to be more active in periportal cells [31,32], but its activity depends on the availability of oxaloacetate and glutamate. The availability of the latter, as already mentioned, should not be a limiting factor. Oxaloacetate, however, could be a limiting factor in periportal cells. This could happen, for example, when a high gluconeogenic activity combines with a relatively reduced state of the malate dehydrogenase reaction, which is detrimental to the oxaloacetate concentration.

Although the glutamine-derived ureogenic flux did not predominate in periportal cells at low ammonia concentrations, this was not determined by the rate of glutamine transformation, which was more elevated in the periportal region for all ammonia concentrations. The latter is indicated by four parameters, ^14^CO_2 _production from labeled glutamine, gluconeogenesis, lactate production and the corresponding oxygen uptake increments which, when normalized with reference to the corresponding cell spaces that are accessible to ammonia via the hepatic artery, were all more pronounced in periportal cells. The relative distribution between periportal and perivenous cells, however, was not the same for all parameters. The periportal predominance of gluconeogenesis, for example, was more pronounced than that of ^14^CO_2 _production. This particular observation has at least two causes. The first one is that the difference in ^14^CO_2 _production reflects solely the unequal distribution of the ammonia-dependent glutaminase along the hepatic acinus [7], whereas the difference in gluconeogenesis reflects both the unequal distributions of glutaminase and of several enzymes of the gluconeogenic pathway which also predominate in the periportal region [2]. The second cause could be related to the inhibitory effect of ammonia on gluconeogenesis. Ammonia is indispensable for glutamine transformation when the latter compound is present at low concentrations, but it also inhibits gluconeogenesis [12,14,15]. Consequently, the final rates of gluconeogenesis in the presence of glutamine plus ammonia are the result of two opposing effects of ammonia: stimulation of glutamine transformation and inhibition of gluconeogenesis. The inhibitory effect of ammonia on gluconeogenesis is not the same in periportal and perivenous hepatocytes [12]. In the present work, indication that it can be more pronounced in perivenous cells is the observation that ammonia infusion into the hepatic artery in antegrade perfusion was always followed by a short period of inhibition of glucose production before stimulation took place (Fig. 3), a phenomenon that was not observed in retrograde perfusion.

## Conclusions

It can be concluded that, at least under the conditions of the present work, the glutaminase activity in periportal hepatocytes is not the rate-controlling step of the glutamine-derived nitrogen flow through the urea cycle [10]. The current view of the hepatic ammonia-detoxifying system proposes that the small perivenous fraction of glutamine synthesizing perivenous cells removes a minor fraction of ammonia that escapes from ureogenesis in periportal cells [3]. It certainly continues to be a valid assumption that the perivenous cells immediately surrounding the hepatic venules, which contain glutamine synthetase and do not contain carbamoyl-phosphate synthase, are able to remove ammonia solely by glutamine synthesis [26,27]. However, ureogenesis can be very active in cells situated downstream to the periportal zone. Under some conditions it can even be more active in these cells than in the periportal cells [12]. This set of observations indicates that ureogenesis is also an important ammonia-detoxifying mechanism in the perivenous region excepting only the small fraction of cells deprived from carbamoyl-phosphate synthase.

## Competing interests

The authors declare that they have no competing interests.

## Authors' contributions

JFC designed and performed the experiments, analysed the data, and drafted the manuscript. FSK co-designed and co-performed the experiments and participated in the discussion of the experimental results. JC co-performed the experiments and participated in the discussion of the experimental results. AB conceived the study, coordinated the implementation of the study and revised the final manuscript.
